# Relationship between CYP3A5 Polymorphism and Tacrolimus Blood Concentration Changes in Allogeneic Hematopoietic Stem Cell Transplant Recipients during Continuous Infusion

**DOI:** 10.3390/ph14040353

**Published:** 2021-04-10

**Authors:** Naoki Yoshikawa, Hidemi Takeshima, Masaaki Sekine, Keiichi Akizuki, Tomonori Hidaka, Kazuya Shimoda, Ryuji Ikeda

**Affiliations:** 1 Department of Pharmacy, University of Miyazaki Hospital, Miyazaki 889-1692, Japan; iamhide3@med.miyazaki-u.ac.jp (H.T.); ryuji_ikeda@med.miyazaki-u.ac.jp (R.I.); 2 Department of Gastroenterology and Hematology, Faculty of Medicine, University of Miyazaki, Miyazaki 889-1692, Japan; masaaki_sekine@med.miyazaki-u.ac.jp (M.S.); keiichi_akizuki@med.miyazaki-u.ac.jp (K.A.); tmnhdk@med.miyazaki-u.ac.jp (T.H.); kshimoda@med.miyazaki-u.ac.jp (K.S.)

**Keywords:** cytochrome P450 family 3 subfamily a member 5, tacrolimus, gene polymorphism, therapeutic drug monitoring, hematopoietic stem cell transplantation

## Abstract

A polymorphism in the gene encoding the metabolic enzyme cytochrome P450 family 3 subfamily A member 5 (CYP3A5) is a particularly influential factor in the use of tacrolimus in Japanese patients. Those who are homozygotic for the *3 mutation lack CYP3A5 activity, which results in substantial individual differences in tacrolimus metabolism. The aim of this study was to analyze the relationship between individual differences in tacrolimus blood concentration changes and *CYP3A5* polymorphisms in allogeneic hematopoietic stem cell transplantation recipients during the period of increasing blood concentration of the drug following treatment onset. This was a prospective observational cohort study, involving 20 patients administered tacrolimus by continuous infusion. The subjects were divided into the *1/*3 and *3/*3 groups based on *CYP3A5* polymorphism analysis. The tacrolimus blood concentration/dose (C/D) ratio increased from day 1 and was largely stable on day 5, and a significant difference was observed between the *1/*3 and *3/*3 groups in the time course of the C/D ratio during this period (*p* < 0.05). This study reveals the effects of *CYP3A5* polymorphism on continuous changes in tacrolimus blood concentration.

## 1. Introduction

Tacrolimus is a macrolide immunosuppressant, and its immunosuppressive activity is mediated by complexes formed with FKBP prolyl isomerase 1A, which binds to calcineurin and inhibits its activity [1,2]. In T cells, downregulating the affected pathways interferes with the nuclear translocation of various factors involved in cytokine transcription [1,2]. Tacrolimus is a prophylactic for post-transplant organ rejection and graft-versus-host disease (GVHD) after allogeneic hematopoietic stem cell transplantation (HSCT), and a therapy for autoimmune disease. HSCT is used to cure blood and hemopoietic tissue abnormalities, such as hematological cancers. Although HSCT is effective, some patients experience GVHD after treatment, which remains a major cause of non-relapse mortality.

A correlation between blood tacrolimus concentration and its clinical efficacy and toxicity as a GVHD prophylactic has been reported [3]. However, the therapeutic range of the blood tacrolimus concentration is narrow, and tacrolimus pharmacokinetics are highly variable among individuals [4,5]. Therefore, therapeutic drug monitoring is required for tacrolimus use. Whole blood is commonly used to assess therapeutic tacrolimus concentrations, as tacrolimus is distributed mainly in red blood cells (RBCs) [6,7]. We examined this characteristic of tacrolimus and reported that differences in RBC distribution are involved in individual differences in tacrolimus blood concentration changes [8,9]. Improving the prediction accuracy of blood concentration changes has a high probability of improving tacrolimus treatment results. Furthermore, pharmacogenomics can also be useful in predicting blood concentration changes [10,11]. Proteins affecting pharmacokinetics can differ at the genetic level in their expression and strength of function, and genetic polymorphisms related to the pharmacokinetics of tacrolimus have been reported [12]. A polymorphism in the gene encoding the metabolic enzyme cytochrome P450 family 3 subfamily A member 5 (CYP3A5) is particularly influential on the effects of tacrolimus in Japanese patients. Tacrolimus is metabolized by CYP3A4 and CYP3A5; however, those homozygous for the single nucleotide polymorphism (SNP) 6986A>G (*3) in intron 3 do not express CYP3A5 [13,14]. It has been reported that approximately 60% of Japanese patients are CYP3A5 *3/*3 [15]. This genetic predisposition causes extremely large individual differences in tacrolimus metabolism ability.

Individual differences in drug metabolism can substantially contribute to drug accumulation in the body, encourage drug–drug interactions (DDIs), and disrupt blood concentration control. Owing to the complexity of the factors influencing tacrolimus pharmacokinetics in HSCT recipients, elucidating the relationship between *CYP3A5* polymorphisms and tacrolimus blood concentration changes is important. In this study, the relationship between individual differences in tacrolimus blood concentration changes and *CYP3A5* polymorphism was analyzed in HSCT recipients, focusing on the period of increasing blood concentration of the drug at the start of administration, during which drug accumulation strongly contributes to treatment efficacy.

## 2. Results

### 2.1. Patient Characteristics

Nine subjects comprised the *1/*3 group, whereas 11 patients comprised the *3/*3 group. The genotype distribution of polymorphisms was consistent with the Hardy–Weinberg equilibrium (X^2^ = 1.31, *p* = 0.5185). Table 1 shows the characteristics of patients in each group at the time of transplantation. There were no significant differences in sex, age, height, body weight, disease diagnosis, transplant conditions, pre-transplant conditioning regimen, clinical laboratory data, or medication use between the groups. There were no pre-emptive dosing changes due to the DDIs, such as aprepitant and amlodipine, in the patients.

### 2.2. Effect of the CYP3A5 Polymorphism on Tacrolimus Pharmacokinetics

**Figure 1:**
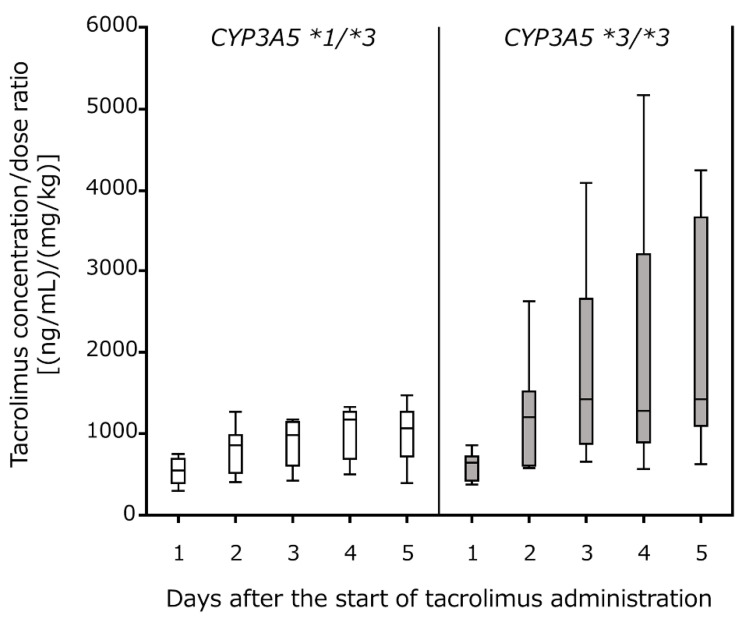
*CYP3A5* genotype-based comparison of tacrolimus concentration/dose ratios during the first 5 days after hematopoietic stem cell transplantation. White (CYP3A5 *1/*3, n = 9) and gray (CYP3A5 *3/*3, n = 11) boxes indicate the median and interquartile range. Vertical lines above and below the boxes indicate the minimum and maximum values.

We analyzed the effects of the *CYP3A5* polymorphism on changes in tacrolimus blood concentration at the beginning of administration. The concentration/dose (C/D) ratio increased from day 1 and was largely stable by day 5 (Figure 1). A significant difference was observed between the *1/*3 and *3/*3 groups in the time course of the C/D ratio during this period (repeated-measures ANOVA, *p* = 0.045). After day 3, the C/D ratio in the *3/*3 group was significantly higher than that in the *1/*3 group on all days (Table 2).

We also analyzed the effect of the *CYP3A5* polymorphism on the changes in the C/D ratio owing to the concomitant use of drugs that interact with tacrolimus metabolism. The combined use of itraconazole, lansoprazole, and letermovir did not produce statistically significant effects; however, the *3/*3 group tended to have higher (C/D)_After_/(C/D)_Before_ ratio than the *1/*3 group after these treatments (Table 3).

## 3. Discussion

In this study, we clarified the relationship between individual differences in tacrolimus blood concentration changes and *CYP3A5* polymorphisms after starting continuous infusion in allogeneic HSCT recipients. Furthermore, we demonstrated that *CYP3A5* polymorphism may cause changes in tacrolimus blood concentration induced by interactions with drugs frequently used concomitantly in HSCT recipients. Several studies have evaluated the effect of *CYP3A5* polymorphism on the changes in blood concentrations at the onset of administration [16,17]. However, most were retrospective, and contained incomplete data regarding the period of greatest blood concentration variability. By prospectively planning our observational study, we were able to incorporate the blood concentrations of all patients for 5 days after the start of tacrolimus in the analysis. As a result, this study revealed the effects of *CYP3A5* polymorphism on the continuous changes in tacrolimus blood concentration with high accuracy for the first time.

The frequency of *CYP3A5* polymorphism in this study was consistent with that in the Japanese population [15]. After day 3, the C/D ratio in the *3/*3 group was significantly higher than that in the *1/*3 group; however, the variation was large. Tacrolimus pharmacokinetics are affected by a variety of factors, especially its absorption and metabolism [18]. However, as the pharmacokinetics of a 24-hour continuous infusion were analyzed, the potential influence of the absorption process was considered extremely small. In other words, in the *3/*3 group, there may be metabolism-related factors that contribute to individual differences in tacrolimus pharmacokinetics in the metabolic process. In *1/*3 heterozygotes, tacrolimus is metabolized by CYP3A5 and CYP3A4; however, in *3/*3 homozygotes, tacrolimus metabolism relies solely on CYP3A4. Therefore, in these people, variations in the CYP3A4 activity may have resulted in variations in the C/D ratio. *CYP3A4* also has several polymorphisms; however, they have not shown significant functional effects in vivo [19]. Furthermore, polymorphisms in cytochrome p450 oxidoreductase (*POR*) have been recently reported as factors related to individual differences in the CYP ability [20,21]. *POR* polymorphisms have been reported to both increase and decrease CYP3A4 activity [22]. That is, in the metabolism of tacrolimus, the effect of *POR* polymorphism on the variations in the metabolism ability of CYP3A5 non-expressers may be more remarkable than CYP3A5 expressers. Therefore, *3/*3 homozygotes could have significant variations in the C/D ratio by *POR* polymorphism. The dependence of *3/*3 homozygotes on CYP3A4 for tacrolimus metabolism may also explain why the effects of the concomitant use of drugs interacting with tacrolimus metabolism appeared stronger in the *3/*3 group than in the *1/*3 group. Aprepitant, which is used for supportive care in conditioning regimens, interacts with tacrolimus *via* CYP3A4. It was used in similar proportions in the two groups in this study and, importantly, it has been reported that aprepitant’s interaction effect disappears rapidly after discontinuation [23,24]; therefore, we believe aprepitant use had a negligible effect on our analysis.

CYP3A5 *6 and CYP3A5 *7 are also important SNPs in tacrolimus dosing. In the *6 and *7 alleles, CYP3A5 is not expressed as in the *3 allele [25]. That is, in the presence of these alleles, the CYP3A5 activity is reduced. The frequency of the *6 and *7 alleles in Japanese and Asians is extremely rare [25,26], but it is considered that these SNPs cannot be ignored. The genotyping of these SNPs should be considered in future studies.

This study has some limitations. First, it was conducted in a single facility, and the sample size was small. Therefore, it is possible that a bias in medical treatment policy affected adverse event control and GVHD expression. Second, the effects of *POR* polymorphisms and their interactions with other patient characteristics were not completely analyzed. It might be necessary to investigate whether *CYP3A5* polymorphisms are independent influencing factors using multivariate analysis. Additionally, larger-scale intervention studies are needed to resolve these issues. Despite these limitations, this study demonstrated the important relationship between *CYP3A5* polymorphism and tacrolimus pharmacokinetics in HSCT recipients, which will be especially useful when establishing initial doses. 

## 4. Materials and Methods

### 4.1. Study Participants

**Figure 2:**
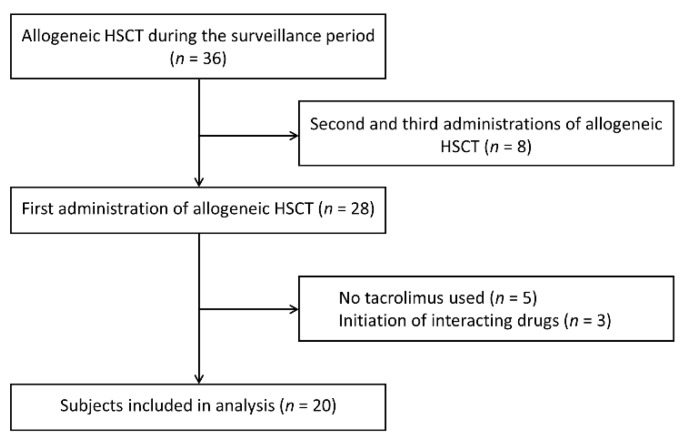
Screening of subjects based on the exclusion criteria. HSCT, hematopoietic stem cell transplantation.

Patients in this study underwent their first allogeneic HSCT in the Department of Hematology at the University of Miyazaki Hospital between January 2018 and October 2020 and were administered tacrolimus (Prograf^®^ Injection, Astellas, Tokyo, Japan) by continuous infusion for GVHD prophylaxis. Patients who received drugs that strongly inhibit or induce CYP3A4 at the start of tacrolimus administration were excluded from the study (Figure 2). In total, 20 patients were enrolled. All participants provided written informed consent before performing any procedure.

### 4.2. Study Design

This was a prospective observational cohort study. Its procedure was approved by the ethics review committee of the Faculty of Medicine at the University of Miyazaki, Japan (G-0036).

After obtaining consent, blood was collected from eligible patients for *CYP3A5* polymorphism analysis. After pre-transplant conditioning treatment, the patients started a 24-h continuous infusion of tacrolimus for GVHD prophylaxis the day before transplant. Only if post-transplantation cyclophosphamide (PTCY) was used to prevent GVHD, patients were started on 24-h continuous-infusion tacrolimus 5 days after HSCT.

The day after the start of tacrolimus administration was set as day 1, and whole blood tacrolimus concentration was measured at 6:00 am daily on days 1–5. Whole blood tacrolimus concentration after day 6 was measured at a frequency determined by the attending physician. Whole blood tacrolimus concentration was measured using the ARCHITECT i1000SR system (Abbott, Tokyo, Japan). Tacrolimus treatment by 24-h continuous infusion was continued for approximately 40 days after HSCT, and then it was switched to oral administration.

The observation period extended from the date of HSCT to the end of tacrolimus infusion or 40 days after HSCT, whichever came first. Patient information (age, sex, height, body weight, clinical laboratory data, and medication) during the observation period and medical histories (disease diagnosis, pre-transplant conditioning regimen, and transplant conditions) were collected from medical records.

### 4.3. CYP3A5 Polymorphism Analysis

To genotype the *CYP3A5* 6986A>G (*3) allele, the polymerase chain reaction-restriction fragment length polymorphism method was used, as previously reported [26]. The results were confirmed using a fully automated SNP detection system (i-densy™ IS-5320, Arkray Inc., Kyoto, Japan). Whole blood collected from subjects before pre-transplant conditioning treatment was used for analysis.

### 4.4. Statistical Analysis

The distribution of alleles in accordance with the Hardy–Weinberg equilibrium was calculated using Chi-squared test. Based on the *CYP3A5* polymorphism analysis, the subjects were divided into *1 (wild-type) allele-bearing (*1/*3) and non-bearing (*3/*3) groups. None of the subjects were homozygous for the *1 allele. The groups were compared in terms of their characteristics at the time of transplantation. Fisher’s exact test was used for nominal variables and Mann–Whitney *U* test was used for continuous variables. Tacrolimus blood concentration (ng/mL) from days 1–5 was divided by the dose per body weight (mg/kg) to calculate the C/D ratio. The mean of the C/D ratio for each day was compared between the *1/*3 and *3/*3 groups using Welch’s *t*-test. In addition, the C/D ratio changes from days 1–5 were compared by repeated-measures analysis of variance (ANOVA). 

The effects of DDIs with concomitant drugs on tacrolimus blood concentration during the observation period were evaluated by dividing the C/D 5–7 days after concomitant use by the C/D 0–2 days before concomitant use ((C/D)_After_/(C/D)_Before_).

R v.4.0.3. (R. Available online: https://www.r-project.org/, accessed on 8 March 2021) was used for statistical analysis. *p* < 0.05 indicated statistical significance.

## 5. Conclusions

The pharmacokinetics of tacrolimus is influenced by not only genetic predisposition but also various factors such as patient’s physiological function and DDI, and individual differences are extremely large. In this study, we found *CYP3A5* polymorphism is an important factor that determines the changes in tacrolimus blood concentration at the start of administration. In other words, one of the factors that enable to predict the changes in blood concentration is *CYP3A5* polymorphism. Although the clinical use of *CYP3A5* polymorphism contributes to tacrolimus dosing determination, it cannot predict the effects of other pharmacokinetic variables. Therefore, a combination of *CYP3A5* genotyping and close blood concentration monitoring is the most effective strategy for precise tacrolimus dosing.

## Figures and Tables

**Table 1:** Patient characteristics.

Characteristics	*1/*3	*3/*3	*p* Value
Number of patients (male/female)	9 (4/5)	11 (9/2)	0.160 ^(a)^
Median age (min–max) (years)	55 (38–63)	61 (38–98)	0.102 ^(b)^
Body weight (kg)	58.6 ± 6.1	59.7 ± 10.3	0.732 ^(b)^
Disease diagnosis			0.610 ^(a)^
AML	1	3	
ALL	1	2	
CML	1	1	
MDS	1	3	
NHL	0	1	
ATL	3	1	
DLBCL	1	0	
MF	1	0	
Stem cell source			0.070 ^(a)^
BMT	8	5	
CBT	0	4	
PBSCT	1	2	
Conditioning regimen			0.281 ^(a)^
Flu/Mel/TBI	4	7	
Cy/TBI	4	1	
Bu/Cy	1	3	
Combination of aprepitant	6	7	1.000 ^(a)^
GVHD prophylaxis			0.056 ^(a)^
Tacrolimus + sMTX	9	6	
Tacrolimus + MMF	0	4	
PTCY + tacrolimus + MMF	0	1	
Laboratory data			
ALT (U/L)	12.9 ± 5.3	27.4 ± 21.5	0.063 ^(b)^
AST (U/L)	13.7 ± 5.5	19.8 ± 6.6	0.055 ^(b)^
GGTP (U/L)	39.3 ± 16.4	46.0 ± 42.9	0.788 ^(b)^
Serum creatinine (mg/dL)	0.54 ± 0.16	0.70 ± 0.19	0.083 ^(b)^
Urea nitrogen (mg/dL)	15.5 ± 2.9	16.6 ± 8.3	0.968 ^(b)^
RBC (10^6^/µL)	2.61 ± 0.47	2.76 ± 0.67	0.941 ^(b)^
Hematocrit (%)	24.6 ± 4.3	26.2 ± 6.2	0.824 ^(b)^
Receipt of amlodipine	1	2	1.000 ^(a)^

AML, acute myeloid leukemia; ALL, acute lymphoblastic leukemia; CML, chronic myelogenous leukemia; MDS, myelodysplastic syndrome; NHL, non-Hodgkin lymphoma, ATL, adult T-cell leukemia-lymphoma; DLBCL, diffuse large B-cell lymphoma; MF, myelofibrosis; BMT, bone marrow transplantation; CBT, cord blood transplantation; PBSCT, peripheral blood stem cell transplantation; Flu, fludarabine; Mel, melphalan; TBI, total body irradiation; Cy, cyclophosphamide; Bu, busulfan; GVHD, graft-versus-host disease; sMTX, short methotrexate; MMF, mycophenolate mofetil; PTCY, post-transplant cyclophosphamide; ALT, alanine aminotransferase; AST, aspartate aminotransferase; GGTP, gamma-glutamyl transpeptidase; RBC, red blood cell. ^(a)^ Fisher’s exact test; ^(b)^ Mann–Whitney U test.

**Table 2:** Tacrolimus concentration/dose ratios during the first 5 days after hematopoietic stem cell transplantation (HSCT).

Genotypes	Day 1	Day 2	Day 3	Day 4	Day 5
*1/*3 (*n* = 9)	540.1 ± 157.2	798.4 ± 283.8	869.3 ± 280.3	988.5 ± 316.3	1000.9 ± 339.7
*3/*3 (*n* = 11)	608.9 ± 166.7	1222.4 ± 676.3	1773.9 ± 1148.8	2018.1 ± 1494.8	2007.6 ± 1367.2
*p* Value	0.356	0.080	0.028 *	0.048 *	0.037 *

Data are mean ± standard deviation [(ng/mL)/(mg/kg)]. *, *p* < 0.05 by Welch’s *t*-test.

**Table 3:** Effects of drug–drug interactions on the tacrolimus concentration/dose ratio.

Drug	Genotype	(C/D)_After_/(C/D)_Before_	Average	*p* Value
Itraconazole	*1/*3	1.11	1.12	-
Itraconazole	*1/*3	1.13		
Itraconazole	*3/*3	1.45	1.55	
Itraconazole	*3/*3	1.59		
Itraconazole	*3/*3	1.61		
Lansoprazole	*1/*3	0.86	-	-
Lansoprazole	*3/*3	1.93	-	
Letermovir	*1/*3	1.29	1.71	0.345 ^(a)^
Letermovir	*1/*3	1.44		
Letermovir	*1/*3	1.73		
Letermovir	*1/*3	1.82		
Letermovir	*1/*3	2.24		
Letermovir	*3/*3	1.41	2.28	
Letermovir	*3/*3	1.52		
Letermovir	*3/*3	2.05		
Letermovir	*3/*3	2.12		
Letermovir	*3/*3	4.31		

C/D, tacrolimus concentration/dose ratio ^(a)^ Welch’s *t*-test.

## Data Availability

The data presented in this study are available on request from the corresponding author.
